# The Influence of Vacuum Impregnation on Nutritional Properties of Fluidized Bed Dried Kale (*Brassica oleracea* L. Var. *Acephala*) Leaves

**DOI:** 10.3390/molecules23112764

**Published:** 2018-10-25

**Authors:** Marta Pasławska, Agnieszka Nawirska-Olszańska, Bogdan Stępień, Angelika Klim

**Affiliations:** 1 Institute of Agricultural Engineering, Wroclaw University of Environmental and Life Sciences, Chełmońskiego Street 37a, 51-630 Wrocław, Poland; bogdan.stepien@upwr.edu.pl (B.S.); angelika.klim@wp.pl (A.K.); 2 Department of Fruit, Vegetable and Plant Nutraceutical Technology, Wroclaw University of Environmental and Life Sciences, Chełmońskiego Street 37, 51-630 Wrocław, Poland; agnieszka.nawirska-olszanska@upwr.edu.pl

**Keywords:** kale leaves, antioxidant activity, vacuum impregnation, fluidized bed drying

## Abstract

The aim of the work was to assess the possibility of obtaining high bioactivity dried kale using a vacuum impregnation as the preliminary processing before the drying. Kale leaves underwent vacuum impregnation in freshly squeezed onion juice and in sodium chloride solution utilising the following impregnation process parameters: At the vacuum stage, 6 kPa reduced pressure for 1 min, dosing the impregnating solution and keeping the sample under vacuum for 2 min, and then 6 min in impregnating solution at atmospheric pressure. Fluidized bed drying of kale was conducted using inert polypropylene balls, utilising a drying air temperature in a range from 70 to 130 °C. The drying kinetics were described, and the dehydrated product’s quality was assessed, on the basis of these selected characteristics: The content of chlorophylls, polyphenols and carotenoids, and antioxidant activity measured with ABTS^+^, dry matter, water activity and colour. It was determined that protective influence of vacuum impregnation before fluidized bed drying was seen only in the case of using temperatures of 90 and 110 °C. The highest content of bioactive components in dried kale was obtained in the case of using onion juice impregnation and drying at 110 °C.

## 1. Introduction

Kale (*Brassica oleracea* L. var. *acephala*) is considered to be the oldest of all *Brassicaceae*. The edible part are its large, curled leaves which have a green or red-purple colour depending on the variety [1]. Despite the fact that kale is a very beneficial vegetable and has proven nutritional and medical properties, it is still underappreciated and less frequently used in European cuisine than other *Brassicaceae*. This vegetable is recommended for the prevention and treatment of cardiovascular diseases, osteoarthritis and immunological system diseases [2], due to its numerous phytonutrients such as vitamins (carotenoids, vitamin C, vitamin E and vitamin K), as well as macro- and microelements (potassium, calcium and iron), secondary metabolites (polyphenols) and dietary fibre [3,4,5]. Kale contains the largest amount of antioxidants of all *Brassicaceae* [6], primarily glucosinolates, a compounds with strong antitumour properties [7]. However, antioxidants are easily destroyed compounds with significant sensitivity to high temperatures and oxygen [8]; therefore, any processing method used should ensure that their levels are maintained to the highest possible degree. Kale, once it has been gathered, has a short shelf-life, which is why it is mainly used for the production of juices and smoothies, as well as an addition to meals after short blanching. It can also be packed in a modified atmosphere, either whole or chopped. However, the most long-lasting form is kale leaves from which water has been removed through drying. Dried kale can be used in products intended for immediate consumption, e.g., dried chips, or as an ingredient of ready-to-eat vegetable mixes, soups and sauces, where it can be the main ingredient of the product or a co-ingredient with others, e.g., carrots [9].

Due to the fact that kale drying should be conducted in a short period of time and at a relatively low temperature, certain existing drying methods may be used, such as drying in a fluidized bed. Fluidized bed drying finds use in the food and chemical industries, since it involves the dynamic exchange of heat and matter [10]. Intensification of the fluidized bed drying process may be achieved through using the highest possible temperature and drying air speed, as well as using inert material that supports the biological material’s fluidization [11,12]. High relative air humidity or high pressure, as well as particles that are too large, slow down the fluidized bed drying process and decrease drying efficiency [13].

Drying dynamics improvement and a high quality of the dehydrated product can be achieved by using initial processing of the biological material, such as osmotic dehydration. During osmotic dehydration, water is removed from the material, while simultaneously, the osmotic substance—a hypertonic solution, usually made of salt or sugar—infiltrates the tissue [14]. Due to consumers’ habits, salt is used for vegetables, while sugar is used for fruit [15]. Osmotic dehydration parameters should be adjusted to the biological material, taking into account the material’s tissue structure and chemical components. The correct osmotic solution concentration and type, process temperature, degree of material fragmentation and osmotic dehydration duration should be selected [16,17,18].

Vacuum osmotic dehydration (vacuum impregnation VI) leads to some advantages as compared with atmospheric osmotic dehydration, because it influences the kinetics of the mass transfer phenomena [19]. Vacuum impregnation consists of conducting osmotic dehydration under decreased pressure and is widely used method of fruit and vegetables pre-treatment before drying [20]. However, the main purpose of VI is incorporating additives into tissue, such as anti-browning agent or minerals.VI is usually made up of two stages: a vacuum stage and an impregnation stage. At the vacuum stage, vegetables or fruit are subjected to reduced pressure, and as a result, most of the air gathered in the intercellular canals is removed until a mechanical balance is achieved [21]. Under conditions of reduced pressure, the material comes into contact with the impregnating liquid, and then atmospheric pressure is restored, while a solution of various components penetrates the material’s porous surface until a balanced pressure is achieved (impregnation stage) [22]. There is a large amount of literature focused on effects of VI conditions on mass transfer phenomena or structure and mechanical properties of impregnated material [20,21,22,23,24,25]. However, little is known about the chemical modifications induced by pressure changes and/or impregnated molecules influencing nutritional properties of processed vegetables.

Thus, the objective of the paper was to evaluate the option of using vacuum impregnation before drying of kale, as an initial procedure ensuring an improvement in drying results and a high quality of the dehydrated product. Onion juice with an addition of sodium chloride and sodium chloride solution were selected as impregnating liquids.

## 2. Results

### 2.1. Nutritional Properties of Dried Kale

The influence of the utilised initial processing on kale nutritional properties: antioxidant capacity and the total content of chlorophyll, carotenoids, and polyphenols in the dehydrated products was evaluated. The influence of both vacuum impregnation (NaCl and O + NaCl) and temperature during fluidized bed drying in the 70–130 °C range on the examined characteristics was confirmed (Table 1).

The kale used in the tests was initially characterised by a total chlorophyll level of 832.64 mg/100 g d.m.; however, during VI, in the case of both O + NaCl and NaCl, as a result of leakage of water with cell components, 11–12% chlorophyll leaked out. Drying non-impregnated material (R) caused significant chlorophyll content decrease, the highest at 70 °C (by 87.5%) and the lowest at 110 °C (by 46%). The influence of impregnation on the chlorophyll content in the dehydrated material was considered positive only when the drying temperature equalled 90 and 110 °C, when no chlorophyll degradation was noted in either case. At other drying temperatures, an intense degradation of the green colourant followed—at 70 °C due to the long drying time and at 130 °C due to the overly high temperature of the process. Considering the influence of the performed actions on chlorophyll content, the most beneficial seems to be the variant in which the initial vacuum impregnation (both in O + NaCl and NaCl) was used and the drying temperature equalled 110 °C.

The carotenoid content in fresh kale leaves equalled 140.06 mg/100 g d.m. It was determined that kale VI in O + NaCl did not result in significant carotenoid content decrease, while after impregnation in NaCl, their content decreased. Drying kale R and O + NaCl caused carotenoid content to decrease, the greatest decrease being at 130 °C (decrease of 55.5% for R and 33% for O + NaCl) and the least reduction at 110 °C (decrease of 4%). The carotenoid content in dehydrated product impregnated in NaCl decreased insignificantly in comparison to the material immediately after impregnation, and it did not depend on drying temperature within the 70–110 °C range. At 130 °C, a degradation of the colourant compounds of the group by 39% was noted.

In the conducted experiments, the polyphenols content in kale did not change as a result of impregnation; however, it decreased significantly as a result of drying. In all experiment variants, the highest polyphenols decrease was noted at 70 °C and the lowest at 110 °C. The positive influence of impregnation on the polyphenols content in dried kale was also noted. Impregnation with NaCl allowed 34–55% of the compounds of this group to be preserved, impregnation with O + NaCl gave 31–51%, while in dehydrated products without initial processing (R) the level of polyphenol preservation equalled 27–42%, depending on the drying temperature.

Product antioxidant capacity results from the presence of antioxidants—various chemical compounds that hinder the reaction of other compounds or macromolecules with oxygen or ozone. The influence of drying temperature on dried kale bioactivity was determined, and in all cases the lowest ABTS^+^ values were noted when the drying temperature equalled 70 °C, while the highest values were noted at 110 °C. This results also prove the positive influence of impregnation on the bioactivity of kale, particularly the sample dried at 110 °C. Admittedly, impregnation with NaCl caused the antioxidant capacity to decrease by 20% and drying decreased it by another 12%; however, during impregnation in O + NaCl a significant decrease in antioxidant activity did not occur, and in the dehydrated product the ABTS^+^ values decreased by only 4.3%. Due to the option of maintaining high oxidative capacity, drying at 110 °C after impregnation in onion juice with salt is regarded as the most beneficial method of kale processing.

### 2.2. Dry Matter Content, Water Activity and Colour of Dried Kale

The initial dry matter content in fresh kale equalled 17.51% (Table 2). VI in onion juice did not change this parameter, while impregnation in salt solution decreased the dry matter content to 13.42%. Therefore, the leakage of soluble components was more intense than salt solution penetration.

Kale dried without impregnation was characterised by a slightly higher dry substance content after drying than impregnated kale. The influence of drying air temperature on dried kale dry matter, regardless of using vacuum impregnation as an initial processing procedure, was confirmed. The highest dry matter content was from non-impregnated kale dried at 130 °C, and the lowest from kale impregnated in onion juice and dried at 70 °C.

VI did not cause the change of the water activity of the material (Table 2), whereas the dried kale was characterised by a water activity level of A_w_ = 0.1837 − 0.4011. The higher the drying temperature used, the lower the water activity level. The dehydrated product obtained from kale impregnated in sodium chloride solution had a much lower water activity than that impregnated in onion juice.

Kale vacuum impregnation in the selected solutions caused value changes to all the colour parameters (Table 3). The material brightened, more in the case of NaCl than O + NaCl. Impregnation also caused decreases in green colour content, visible as increases in parameter a* values (in the case of both impregnating solutions) and decreases in yellow colour content, visible as decreases in b* parameter values, higher in the case of NaCl than O + NaCl. The results confirm the correct course of vacuum impregnation and the occurrence of anti-directional matter transport shown by the leakage of water with plant colourants (chlorophyll and carotenoids) and the penetration of impregnating ingredients into tissues. It was particularly visible in the case of changes in the b* parameter, when colourant compounds transferred from the onion caused an increase in the yellow colour content in comparison to kale impregnated with sodium chloride solution. After the vacuum impregnation, a decrease in colour hue h* was also noted, higher in the case of O + NaCl impregnation than NaCl impregnation, and a decrease in colour saturation C*, higher in the case of NaCl usage. A confirmation of the high degree of colour change caused by impregnation is the high value of the total colour difference coefficient ΔE, equal to 20.08 for O + NaCl and 31.82 for NaCl.

Kale fluidized bed drying caused a significant change in the examined colour parameters, dependent on the drying temperature and on whether the impregnation was conducted or omitted. In non-impregnated samples, a brightening up of the materials after drying was noted (L* parameter value decrease), the highest being at 70 °C, the lowest at 130 °C. On the other hand, the impregnated samples darkened in comparison to their pre-impregnation state, the most visible one being kale impregnated in NaCl and dried at 110 °C. A similar effect was noted in the case of the a* and b* parameters. In non-impregnated (R) samples, an a* parameter value increase was noted, while in impregnated samples, the a* parameter value decreased, most notably at 110 °C. The results obtained confirm the possibility of protecting the green colourant from thermal degradation through covering plant tissues with impregnating solution. Eventually, the green colour content in initially impregnated dehydrated products became equal to the green colour content in non-impregnated dehydrated products, which means that the change of green colour caused by chlorophyll leakage from the material during impregnation was comparable in size to the colour changes caused by degradation of the colourant during thermal processing.

Dried kale colour hue h* and saturation C* were significantly influenced by the drying temperature. The colour hue of kale dried without initial impregnation decreased by ca. 4 units. Dehydrated product colour saturation differed significantly from fresh material colour saturation, and a dependency of inverse proportion between dehydrated product colour saturation and drying temperature was noted.

On the basis of ΔE parameter, it can be determined that the colour of all the dehydrated materials differed significantly from the colour of the fresh material. The highest value of total colour difference (ΔE = 42.20) between fresh kale and the dehydrated product for all samples was noted in the case of a sample dried at 130 °C without vacuum impregnation. In the case of using impregnation, the range of ΔE value changes between the level before drying and the level after drying was much smaller than in R samples, which indicates the protective influence of both utilised impregnating solutions on kale colour during dehydration.

### 2.3. Drying Kinetics

To describe the fluidized bed drying kinetics of kale leaves, a Page model (Equation (1)) was selected:

(1) $$MR=\exp\left(-k\times \tau^{a}\right)$$

where: *k*—dehydration coefficient [min^−1^]; *a*—model parameter; *τ*—time [min].

The fit of the tested mathematical model to the experimental data was evaluated on the basis of an assessment of the goodness of fit of statistical coefficients (Table 4).

The analysed statistical coefficients indicated the suitability of the Page model for the kale fluidized bed drying kinetics, as proven by the low values of mean square error *RMSE* (0.00178–0.02410), the relatively low values of residual variation coefficients *V_e_* (1.1–9.3), and the low values of reduced test *X*^2^ (0.00002–0.00232).

The angular coefficient of the *k* function (dehydration coefficient) in the Page model changed, depending on whether an impregnating solution was used before dying or not, but the kind of solution did not influence its value. On the other hand, the value of the coefficient *a* increased with temperature increase, both in the case of utilising vacuum impregnation and in samples dried without initial processing.

**Figure 1:**
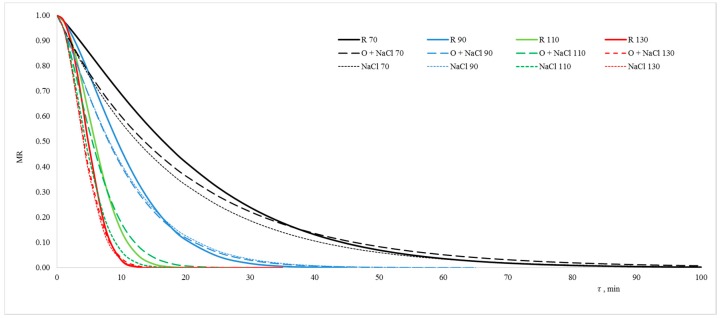
Drying kinetics of kale leaves nonimpregnated (R) and impregnated in onion juice (O + NaCl) or salt solution (NaCl) and spouted bed dried by temperature 70 (black lines), 90 (blue lines), 110 (green lines) and 130 °C (red lines).

The rate of kale dehydration increased along with increasing drying air temperature, both in the case of leaves subjected to vacuum impregnation and non-impregnated ones (Figure 1). Using VI as the initial procedure before drying quickened dehydration in the initial process stage at all utilised temperatures and in the case of both impregnating solutions. During final drying stage, on the other hand, a slowing down of the dehydration was noted as compared to the drying of non-impregnated material, with the exception of kale dried at 110 °C after impregnation in salt solution. The positive influence of impregnation on the material drying rate in the first drying stage was particularly visible at 70 °C, even though dehydration of kale impregnated with sodium chloride solution occurred more quickly than that of kale impregnated with onion juice.

During drying at 90 °C, the influence of impregnation on drying kinetics was visible as well, but the kind of impregnating solution did not influence the dehydration process. Raising the air temperature up to 110 °C resulted in a speeding up of the process at the first stage, but a sample of the material impregnated in salt solution dried much more quickly than the others. The drying of the material at 130 °C occurred very intensely, both in the case of pre-processed and unprocessed samples, and the phenomenon of speeding up the dehydration in the first process stage and slowing it down in the second drying stage was visible in the case of both the utilised impregnating solutions.

## 3. Discussion

Drying process of agricultural materials involves removal of unbound water from the surface (a constant rate evaporation period) and then removal of bound water from the interior (falling rate evaporation period), till an appropriate moisture is reached. Whereas, thermal drying of leaf usually occurs in one stage, as unbound moisture is insignificant in leaves. Various mathematical models have been proposed for describing drying kinetics of different leaves [26]. In presented paper, as the best model describing fluidized bed drying characteristic of kale, Page model was used. Page model was also recommended as the most suitable for modelling of kale [27] and other leaves drying: Thymus [28], mint [29] rosemary [30] and parsley [31].

Vacuum impregnation has been reported as a positive pre-treatment processing before drying apples [23] and tomatoes [24], causing improving in mass transfer dynamics and quality of product. Vacuum impregnation of kale caused increasing in constant rate and decreasing in falling rate of drying. The improvement of drying kinetics in the first process stage resulted from the opening of intracellular canals and the loosening of the kale leaves’ tissue structure caused by using decreased pressure during vacuum impregnation. Meanwhile, the cause of dehydration rate decrease in the material’s second drying stage can be attributed to the presence of the impregnating solution, which slowed down the diffusion of physically and chemically bonded water in the deeper layers of the tissue structure by penetrating them. Therefore, the influence of vacuum impregnation on kale drying kinetics was confirmed. During the osmotic dehydration, the initial material mass decreases due to the removal of water with soluble ingredients. Simultaneously, the impregnating solution causes a sample mass increase by impregnating the material due to capillary suction and diffusion [25]. The slowing down of the dehydration of the kale impregnated in onion juice was larger than in the case of sodium chloride solution, since onion juice contains many more water-soluble ingredients.

Occhino et al. [26] conducted vacuum impregnation of zucchini in maltodextrin solutions, NaCl and CaCl_2_. On the basis of their results they determined that zucchini vacuum impregnation is a beneficial pre-treatment before drying, since it initiates intense dehydration, while simultaneously not causing negative structural changes, and thus allows a high-quality product to be obtained.

During intensive heat and mass transport biological structures of material are exposed to drying conditions: Temperature and relative humidity of air. The typical effect of temperature on kale drying rate was observed. Increased temperature caused increasing the moisture holding capacity of the air and also accelerating the water diffusion. A similar dependency was noted in the case of drying basil leaves [32], rosemary [30] and drumstick leaves [33]. However, during hot air drying, proportionally to increasing temperature, leaves undergo structural and chemical changes that can affect texture, colour and nutritional properties [34]. For many years leaves have been dried at temperature ranging from 40 to 60 °C, as the temperature indicated to cause minimal loss of quality in herbs, spices [35] and medicinal plants [36]. On the other hand, low temperature may encourage decomposition by activating of native enzymes, result in microbial attack and also significantly slows down the drying rate [37]. Therefore, the temperature at which leaves of different types are to be dried has to be selected to sustain nutritional properties. In case of kale leaves dried in fluidized bed (both impregnated and non-impregnated), the temperature of 70 °C was found as to low, adverse due to long lasting process and decomposition of all evaluated nutritional components. The drying temperature of 130 °C was also classified as adverse, because despite the intensive dehydration caused deterioration of quality.

The bioactivity of *Brassicaceae* is determined on the basis of their biologically active compounds content and antioxidant activity. Due to the fact that during preserving at high temperature the content of valuable ingredients usually decreases, dehydration parameters should be selected in order to minimise the loss of biologically active ingredients. The colour of the purchased product is the first feature that the customer pays attention to, and a crucial factor of product attractiveness. The colour parameters of the CIE L*a*b* system were assessed, such as product lightness (L*), chromaticism (a*, b*), hue (h*), and saturation (C*), as well as the total colour difference between the sample and the colour of fresh kale that had not undergone vacuum impregnation (ΔE). The CIE L*a*b* colour classification system allows luminescence (lightness) to be directly assessed using the L* parameter, which equals 0 for white and 100 for black, and chromatic coordinates: a* (+60 is red colour, and −60 green colour) and b* (+60 is yellow colour, and −60 blue colour) [38]. Interpretation of the total colour difference coefficient ΔE was determined by the International Commission on Illumination CIE and is as follows:

- ΔE = 0–2—colour difference is undetectable;
- ΔE = 2–3.5—colour difference is detectable by a human eye;
- ΔE ≥ 3.5—clear colour difference.

The drying process of plant-based products usually leads to the degradation of colourant compounds evident as brightening up (L* parameter value decrease), and in the case of green plants also browning and chlorophyll disintegration—a colour change from green-blue to yellow-red [39], which numerically presents itself as an increase in the value of both chromatic parameters, a* and b*. This dependency proved true in the case of the drying of kale without initial processing; however, it did not do so when vacuum impregnation was utilised (Table 3). Few studies have been performed on the effect of thermal processing on the colour of vegetables. Armesto et al. [40] reported changes in colour parameters of thermal processed kale. They observed darkening of material and the loose of greenness during boiling, cooking and storage of kale. They subjected kale to cooking at normal pressure and various levels of reduced pressure, noting a decrease in chlorophyll content of 36–58%, evaluated likewise decrease of yellowness and colour intensity (C*) but increase of hue angle (0 or 360° = red, 90°= yellow, 180° = green and 270° = blue), what can be a result of carotenoids and chlorophyll degradation.

Chlorophyll is responsible for the green colour of plants, and its presence in vegetables increases their nutritional value—it has antitumour and antimutagenic properties [41]. The kale used in the tests was initially characterised by a total chlorophyll level of 832.64 mg/100 g d.m.; however, during the impregnation, in the case of both O + NaCl and NaCl, as a result of leakage of water with cell components, 11–12% chlorophyll leaked out (Table 1). This is also confirmed by the green colour changes measured by the CIE L*a*b* system presented earlier. Other methods of thermal processing, such as blanching or cooking, decrease chlorophyll content as well. Korus [42] researched the influence of blanching kale before convective drying at 55 °C on total chlorophyll content and noted a decrease of 11%. Araújo et al. [43] also researched the influence of the initial processing and drying conditions on the chlorophyll content of kale. They determined that with an increase in the convective drying temperature within a 40–70 °C range, the chlorophyll content in the dried, initially blanched with water vapour (under 101.325 Pa pressure for 1 min) kale decreased by 38–60%. The difference between the results of drying at 70 °C is noteworthy—it possibly results from the fact that the final dry matter content of kale convectively dried by researchers equalled 25.5%, while the dry matter content achieved in the presented experiments during fluidized bed drying equalled 92.48–94.13% for R. The results achieved by the authors of the paper suggest the possibility of obtaining highly dehydrated material, which therefore would have a long shelf life, and confirm the positive influence of vacuum impregnation on the chlorophyll content in dried kale.

Chlorophyll content changes are closely connected to material colour, since the decomposition of chlorophyll (green colourant) to pheophytin (a compound with an olive-brown colour) appears as a change of chromatic parameters. Carotenoids are natural, preventive and intervening antioxidants, since they hinder free radicals’ activity on the basis of two mechanisms: reduction and oxygenation [44]. Korus [45] demonstrated the positive influence of blanching during convective drying at 55 °C, noting degradation of the compounds of the group by 6%.

Polyphenols, which are classified as secondary plant metabolites, are regarded as an important element of lifestyle diseases prevention due to their antioxidant properties. They have antifungal, anti-inflammatory and antiviral properties, and they decrease the risk of tumours and osteoporosis [46]. The negative influence of thermal processing on polyphenol content in plant-derived materials has been described by numerous authors, who explain the phenomenon by the Maillard reaction [47], polymerisation [48], or oxidation and condensation [49]. Thus, researchers have looked for methods of stopping these processes by initial processing, such as blanching, cooking or undergoing reduced pressure. Korus [45] researched the influence of blanching before convective drying on the polyphenol content of kale leaves. She determined that the blanching process caused polyphenols to degrade by 32%, and convective drying at 55 °C decreased their level by another 33%, while the polyphenol content decrease after drying non-blanched kale equalled 59%. Armesto et al. [40] described the decrease in the polyphenol content of kale leaves undergoing cooking at atmospheric and under reduced pressure. Utilising reduced pressure during cooking decreased polyphenol degeneration from 73% to 16%.

## 4. Materials and Methods

### 4.1. Material

Kale (*Brassica oleracea* L. *var. acephala*), Reflex F1 variety, was purchased retail, stored at 4 °C, and then cleaned, dried off and cut into 1 × 1 cm pieces prior to the tests.

### 4.2. Vacuum Impregnation

**Figure 2:**
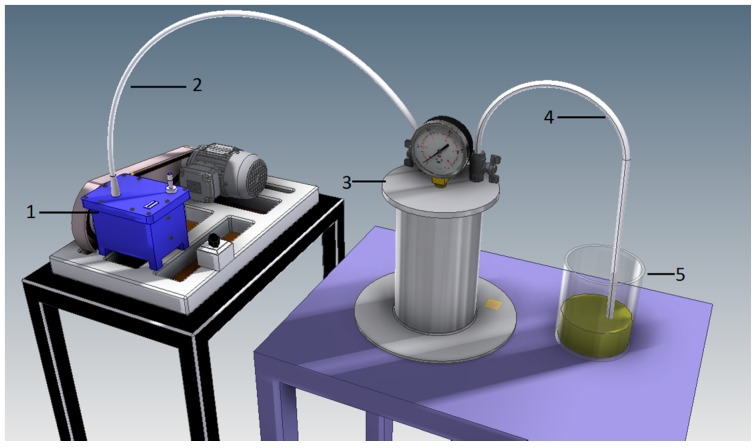
Experimental stage for vacuum impregnation. 1—vacuum pump; 2—vacuum pipe; 3—impregnation vessel; 4—impregnant pipe; 5—impregnation liquid.

The material underwent VI utilising two impregnating solutions: Freshly squeezed onion juice with 1% sodium chloride (O + NaCl), and 1% sodium chloride solution (NaCl). Pressure impregnation was conducted in a prototype installation (Figure 2) located in the laboratory of the Institute of Agricultural Engineering, Wrocław University of Environmental and Life Sciences. The total impregnation time equalled 10 min, by following this procedure: a 100 g kale portion was placed in an impregnation vessel and subjected to reduced pressure 0.06 MPa ( reduction time: 30 s) for 1 min, and then impregnating solution was drawn in and the material kept in it for 2 min. The impregnation stage was conducted for another 6 min after restoring atmospheric pressure (recovery time: 30 s); The impregnating solution was then drained from the kale, which was lightly dried with filter paper.

### 4.3. Drying

One hundred g portions of fresh (R) and impregnated (O + NaCl and NaCl) material underwent drying, and in order to achieve a better fountaining effect and even material heating, polypropylene inert balls of 5 mm diameter were used in a 1:1 mass ratio to the material.

**Figure 3:**
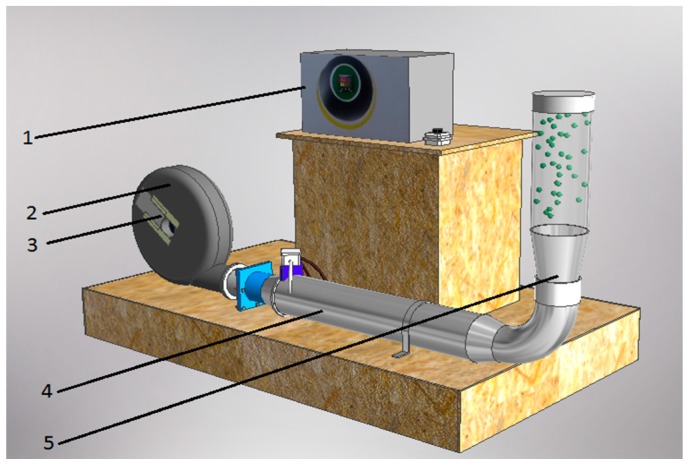
Spouted bed drier. 1—temperature controller; 2—fan; 3—regulatory air shutter; 4—heater; 5—drying chamber.

The drying was conducted in a fluidized bed drying installation (Figure 3), located in the Institute of Agricultural Engineering, Wrocław University of Environmental and Life Sciences (Wrocław, Poland). The drying was conducted at temperatures of 70, 90, 110 and 130 °C, using a drying air speed in the range of 4–10 m/s, adjusted to ensure correct bed fluidization. Dehydration was carried out until a balanced water content was achieved.

The drying kinetics were described on the basis of bed weight loss assessment at 5-min intervals (Radwag WLC 3/6/A2 ± 0.01 g). The tests were repeated three times.

Relative water content was calculated on the basis of Equation (2):

(2) $$MR=\frac{u_{\tau }-u_{r}}{u_{o}-u_{r}}$$

where: *MR*—relative water content [−]; *u_r_*—reduced water content [g H_2_O/g d.m.]; *u_o_*—initial water content [g H_2_O/g d.m.]; *u_τ_*—water content after time *τ* [g H_2_O/g d.m.].

The dried material was vacuum-packed into PA/PE containers (TEPRO PP5.4, TEPRO, Koszalin, Poland; 80% vacuum) and stored at 4 °C. To evaluate the influence of impregnation on the kale drying results, selected quality characteristics of the dehydrated product were used, such as dry matter content, water activity, colour and the content of selected bioactive compounds.

The obtained dehydrated product was evaluated on the basis of selected quality characteristics: dry matter content (thermogravimetric method [50], electronic balance weight AS/160/C/2, Radwag, Radom, Poland, the accuracy of measurement ± 0.0001 g) water activity (AquaLab Dew Point Water Activity Meter 4TE ± 0.003, AquaLab, Warszawa, Poland;) and colour parameters in the CIE L*a*b* system (Minolta CR400, Minolta Conica, Japan), in which the reference sample is white.

On the basis of measurements of L*a*b* parameters, the absolute colour difference between the examined samples and the fresh material (ΔE) was calculated from Equation (3), the colour saturation (C*) from Equation (4), and the hue angle (h*) from Equation (5) [38]:

(3) $$\Delta E=\sqrt{{\left(\Delta L^{*}\right)}^{2}+{\left(\Delta a^{*}\right)}^{2}+{\left(\Delta b^{*}\right)}^{2}}$$

(4) $$C^{*}=\sqrt{{\left(a^{*}\right)}^{2}+{\left(b^{*}\right)}^{2}}$$

(5) $$h^{*}=tg^{-1}\left(\frac{b^{*}}{a^{*}}\right)$$

where: L*—lightness; a*, b*—colour chromatic coordinates; ΔL*, Δa*, Δb*—colour difference between fresh and dried samples.

### 4.4. Chemical Analysis

The total content of carotenoids as well as that of a and b chlorophyll was determined by the colorimetric method (UV-160A spectrophotometer, Shimadzu, Osaka, Japan). 15 mL of 100% acetone was added to a 2 g kale sample and then left for 24 h at 4 °C. The sample then underwent centrifugation at 12500 rpm for 5 min. Absorbance coefficients were measured at wavelengths of 661.6, 644.8 and 470 nm. The contents of carotenoids and chlorophyll a and b in kale were calculated [51].

Polyphenols were tested using the Folin-Ciocalteu method (Shimadzu UV-2401 PC spectrophotometer, Osaka, Japan), in which gallic acid was utilised. The sample absorbance was measured at a wavelength of 765 nm, and the result was expressed in terms of gallic acid (mg gallic acid /100 g dry matter) [52].

The kale’s antioxidant capacity were tested using the ABTS^+^ method (Shimadzu UV-2401 PC spectrophotometer, Osaka, Japan). ABTS^+^ solution was diluted with redistilled water until it reached 0.700 (±0.002) absorbance at a wavelength of 743 nm. 60 µL of supernatant solution was then added to 3 mL of ABTS^+^, and the absorbance measurement was conducted after 6 min [53].

All tests were repeated three times, with the exception of the colour parameters, which were repeated six times.

### 4.5. Statistical Analyses and Software

For the purpose of selecting the mathematical model for the description of the drying kinetics, the following statistical coefficients were calculated: Mean square error *RMSE* (Equation (6)), reduced test values *X*^2^ (Equation (7)), and the residual variation coefficient *V_e_*. (Equation (8)):

(6) $$RMSE=\sqrt{\frac{\sum_{i=1}^{N}{\left(MR_{i,p}-MR_{i,e}\right)}^{2}}{N}}$$

(7) $$X^{2}=\frac{\sum_{i=1}^{N}{\left(MR_{i,p}-MR_{i,e}\right)}^{2}}{N-n}$$

(8) $$V_{e}=\frac{\sqrt{X^{2}}}{Y}\cdot 100\%$$

where: *MR_i,p_*—calculated value of relative water content; *MR_i,e_*—experimental value of relative water content; *N*—number of conducted observations; *n*—number of parameters in the model equation; *Y*—experimental average of relative water content *MR* [54].

To determine the significance of the influence of temperature and impregnating solution type on selected quality characteristics of the dehydrated product, unidirectional bifactor variation analysis was conducted using an Excel 2007 spreadsheet (Microsoft, Redmond, Washington USA). To find distinct homogenous groups, the Tukey test was used. The results were determined on the basis of an average of three repetitions, with an exception of colour parameters, where there were six repetitions instead. The statistical analysis of colour parameters was conducted using Statistica 2012 software (Tibco Statistica, Palo Alto, CA, USA).

The impregnation device outline and drying system layout were prepared using an educational version ofInventor 2018 software (Autodesk Inventor, San Rafael, CA, USA).

## 5. Conclusions

The present study has shown that VI as preliminary processing of kale before drying leads to improvement of nutritional properties, especially antioxidant capacity of dried material. Of the two impregnation liquids (salted onion juice and salt solution) and four temperatures investigated (70, 90, 110 and 130 °C), the smallest antioxidant capacity losses were determined at drying temperature of 110 °C and pre-treatment in onion juice.

## Figures and Tables

**Table 1:** Bioactive components: polyphenols, carotenoids, chlorophylls and antioxidant activity ABTS^+^ of kale leaves spouted bed dried by temperature 70, 90, 110 and 130 °C when nonimpregnated (R) and impregnated in onion juice with salt (O + NaCl) or salt solution (NaCl).

Drying Conditions		Bioactive Components			
Material	T [°C]	Chlorophylls [mg/100 g d.m.]	Carotenoids [mg/100 g d.m.]	Polyphenols [mgGA/100 g d.m.]	ABTS^+^ [µMol/100 g d.m.]
R	-	832.64 ± 12.45 ^a^	140.06 ± 10.23 ^a^	626.17 ± 54.32 ^a^	57.17 ± 2.15 ^a^
	70	103.77 ± 9.35 ^g^	104.21 ± 11.36 ^c,d^	170.06 ± 15.21 ^e^	21.37 ± 1.21 ^g^
	90	203.88 ± 17.36 ^e^	102.86 ± 8.14 ^d^	233.45 ± 12.23 ^c^	28.89 ± 1.36 ^e^
	110	453.33 ± 28.37 ^d^	134.66 ± 9.74 ^b^	268.88 ± 20.54 ^b,c^	34.81 ± 2.25 ^d^
	130	141.44 ± 12.45 ^f^	62.31 ± 4.21 ^f^	240.34 ± 21.25 ^c^	30.84 ± 2.18 ^d,e^
O + NaCl	-	739.21 ± 13.51 ^b^	138.52 ± 11.53 ^a^	658.65 ± 25.41 ^a^	50.12 ± 1.27 ^a^
	70	141.75 ± 11.01 ^f^	133.53 ± 12.65 ^b^	206.04 ± 17.25 ^d^	23.31 ± 1.98 ^f^
	90	723.89 ± 35.01 ^b^	131.47 ± 11.58 ^b^	271.25 ± 21.36 ^b,c^	29.86 ± 1.14 ^e^
	110	729.09 ± 48.14 ^b^	138.21 ± 14.24 ^a^	337.85 ± 29.24 ^b^	47.99 ± 3.25 ^b^
	130	169.79 ± 12.12 ^f^	93.07 ± 6.32 ^e^	306.20 ± 24.12 ^b^	38.11 ± 2.15 ^c^
NaCl	-	732.78 ± 12.54 ^b^	108.08 ± 14.52 ^c^	534.25 ± 17.21 ^a^	45.87 ± 2.54 ^b^
	70	132.83 ± 11.32 ^f^	103.94 ± 13.65 ^d^	183.57 ± 15.87 ^e^	21.60 ± 1.96 ^g^
	90	639.53 ± 35.21 ^c^	102.44 ± 10.29 ^d^	227.50 ± 19.23 ^c^	27.27 ± 1.36 ^e^
	110	723.83 ± 42.79 ^b^	103.44 ± 12.06 ^d^	298.28 ± 21.59 ^b^	40.49 ± 2.34 ^c^
	130	164.96 ± 14.25 ^f^	66.19 ± 2.13 ^f^	285.27 ± 24.24 ^b^	34.42 ± 2.54 ^d^

Values are mean ± standard deviation, n = 3; in columns, mean values with different letters (a, b, c…).

**Table 2:** Dry matter (d.m. [%]) and water activity (A_w_ [−]) of kale leaves spouted bed dried by temperature 70, 90, 110 and 130 °C when nonimpregnated (R) and impregnated in onion juice with salt (O + NaCl) or salt solution (NaCl).

Drying Conditions		Drying Results	
Material	T [°C]	d.m. [%]	A_w_ [−]
R	-	17.51 ± 1.12 ^b^	0.9888 ± 0.0300 ^a^
	70	94.13 ± 1.74 ^d^	0.3831 ± 0.0126 ^b^
	90	95.20 ± 1.56 ^e^	0.3814 ± 0.0026 ^b^
	110	95.55 ± 2.25 ^e^	0.3953 ± 0.0466 ^b^
	130	96.62 ± 1.98 ^f^	0.3620 ± 0.0066 ^c^
O + NaCl	-	17.15 ± 2.00 ^b^	0.9826 ± 0.0010 ^a^
	70	92.48 ± 0.18 ^c^	0.4011 ± 0.0025 ^b^
	90	93.24 ± 0.37 ^c^	0.3704 ± 0.0134 ^c^
	110	94.28 ± 0.69 ^d^	0.3416 ± 0.0609 ^d^
	130	94.74 ± 0.54 ^d^	0.3445 ± 0.0276 ^d^
NaCl	-	13.42 ± 1.07 ^a^	0.9859 ± 0.0033 ^a^
	70	93.79 ± 0.30 ^c^	0.2941 ± 0.0339 ^e^
	90	94.93 ± 0.22 ^d^	0.2401 ± 0.0037 ^e^
	110	94.91 ± 1.37 ^d^	0.1930 ± 0.0030 ^f^
	130	95.76 ± 0.10 ^e^	0.1837 ± 0.0104 ^f^

Values are mean ± standard deviation, n = 3; in columns, mean values with different letters (a, b, c…).

**Table 3:** Colour factors: lightness (L*), greenness (a*), yellowness (b*), hue angle (h*), saturation (C*) and total colour difference (ΔE) of kale leaves spouted bed dried by temperature 70, 90, 110 and 130 °C when nonimpregnated (R) and impregnated in onion juice with salt (O + NaCl) or salt solution (NaCl).

Drying Conditions		Colour Factors					
Material	T [°C]	L*	a*	b*	h*	C*	ΔE
R	-	44.26	−13.09	22.30	−58.8 ^a^	29.11	-
	70	35.13	−6.23	8.79	−60.70 ^c^	10.19	22.14
	90	37.32	−6.27	11.31	−62.96 ^c^	12.92	39.49
	110	37.74	−6.62	10.98	−58.73 ^a^	12.82	39.85
	130	39.35	−7.31	10.97	−62.65 ^c^	15.24	42.20
O + NaCl	-	36.72	−0.63	17.23	−87.90 ^f^	17.24	20.08
	70	38.50	−5.11	10.70	−64.44 ^d^	11.86	8.88
	90	37.75	−6.67	11.29	−58.98 ^a^	13.21	39.95
	110	38.01	−7.37	12.42	−59.29 ^a^	14.45	40.67
	130	38.39	−7.02	13.03	−61.73 ^c^	14.83	41.16
NaCl	-	27.67	−0.96	3.57	−74.91 ^e^	3.69	31.82
	70	31.37	−4.27	7.30	−59.68 ^a^	8.47	6.21
	90	34.79	−6.31	10.80	−59.72 ^a^	12.52	36.98
	110	37.16	−8.85	13.58	−56.90 ^b^	16.22	40.55
	130	32.91	−5.99	9.45	−57.61 ^a,b^	11.20	34.76

**Table 4:** Page model coefficients and statistical coefficients used for mathematical modeling of kale leaves spouted bed dried by temperature 70, 90, 110 and 130 °C when nonimpregnated (R) and impregnated in onion juice with salt (O + NaCl) or salt solution (NaCl).

Drying Conditions		Page Model Coefficients $MR=\exp\left(-k\times \tau^{a}\right)$		Statistical Coefficients		
Material	T [°C]	*k*	*a*	*RMSE*	*V_e_* [%]	*X* ^2^
R	70	0.023	1.2168	0.01325	4.5	0.00070
	90		1.5256	0.01401	4.8	0.00078
	110		1.9278	0.01383	2.1	0.00077
	130		2.1500	0.00311	1.4	0.00002
O + NaCl	70	0.054	0.9800	0.02220	9.3	0.00197
	90		1.2292	0.01728	6.9	0.00119
	110		1.5083	0.01025	3.6	0.00042
	130		1.7855	0.00404	2.5	0.00007
NaCl	70	0.054	1.0087	0.02410	11.2	0.00232
	90		1.2145	0.01658	6.2	0.00082
	110		1.7704	0.00178	1.3	0.00110
	130		1.8077	0.00230	1.1	0.00002
